# Assessment of Quality of Life of Children and Adolescents with Type 1 Diabetes in Bahrain Using PedsQL 3.2 Diabetes Module

**DOI:** 10.3390/jcm14072216

**Published:** 2025-03-25

**Authors:** Aseel AlSaleh, Jamil Ahmed, Intisar Alsenani, Wijdaan Alhousni, Riham AalAbdulsalam, Marya Tawfeek Alshammasi

**Affiliations:** Department of Family and Community Medicine, College of Medicine and Health Sciences, Arabian Gulf University, Manama P.O. Box 26671, Bahrain

**Keywords:** diabetes mellitus, type 1, quality of life, adolescent, child, Bahrain, health-related quality of life

## Abstract

**Background/Objectives**: Living with type 1 diabetes (T1D) significantly impacts children and adolescents, affecting their physical, emotional, and social well-being. Poor glycemic control (HbA1c > 7.5%) is linked to lower health-related quality of life (HRQoL), highlighting the need for effective management. This study aimed to assess the HRQoL and evaluate the associations between various factors and domains of HRQoL among children and adolescents with T1D in Bahrain. **Methods**: This cross-sectional study enrolled 182 children and adolescents from 5 to 16 years with T1D from a tertiary care hospital. Participants had T1D for at least six months and were interviewed during regular follow-ups. Participants Outside the target age group and those with any comorbidity were excluded. Data collection involved demographic and diabetes-related information. The PedsQL 3.2 Diabetes Module was used to assess HRQoL. **Results**: The mean age at diagnosis was 6.83 ± 3.11 years, with 57.7% diagnosed between 6 and 11 years. The sample was gender-balanced (52.2% male, 47.8% female). Treatment adherence had the highest median score (80.0), while worry was the lowest (58.33). Diabetes symptoms were associated with family income, school performance, HbA1c, and emergencies. Treatment barriers were linked to age, education, insulin regimen, and glucometer type. Adherence correlated with age, age at diagnosis, sex, BMI, education, and comorbidities, with family income (β = 4.69, *p* = 0.032) and school performance (β = −22.986, *p* < 0.001) being significant predictors. Treatment adherence was negatively impacted by younger age (β = −20.651 for 6–8 years, β = −12.002 for 9–12 years, both *p* < 0.01) and comorbidities (β = −12.286, *p* = 0.021). **Conclusions**: This study highlights the significant impact of various factors on the HRQoL of children and adolescents with T1D in Bahrain, emphasizing the need for targeted interventions to improve their overall well-being.

## 1. Introduction

Living with type 1 diabetes (T1D) presents unique challenges, especially for children and adolescents who must balance the complexities of managing their condition alongside their academic and social lives. Children with T1D who have poor glycemic control (HbA1c > 7.5%) exhibit lower health-related quality of life (HRQoL), highlighting the necessity for effective management strategies [1]. Measuring HRQoL in children with TID provides a comprehensive understanding of how the condition and its treatment impact their lives and well-being [2]. It goes beyond clinical outcomes to capture the physical, emotional, and social dimensions of health, helping communities struggling with heavy burden of T1D. In the context of T1D, HRQoL assessments can identify areas where children struggle the most, guiding targeted support and resources to enhance their overall health and satisfaction with care [1]. This is particularly important for children, as managing diabetes can significantly affect their school performance, social interactions, and emotional health. By understanding these impacts, healthcare professionals can develop more effective, child-centered care plans that address both medical and psychosocial needs [3].

The daily challenges of T1D among pediatric groups include insulin regimens, dietary restrictions, and physical activity. They also suffer from low self-esteem, anxiety, and depression due to their disease [4]. Additionally, patients with T1D have numerous long-term complications including diabetic retinopathy, diabetic nephropathy, and diabetic neuropathy in pediatric patients [5]. This has led to an increased emphasis on assessing and improving patients’ HRQoL which refers to the impact that a disease or condition has on an individual’s physical, mental, and emotional well-being. As a result, it has become an increasingly significant metric for evaluating medical treatments’ effectiveness [6]. The onset of T1D in children is associated with complications such as poor metabolic control, which can lead to lower HRQoL. Additionally, managing diabetes in children requires comprehensive healthcare resources, including regular monitoring, insulin therapy, and education on lifestyle modifications.

HRQoL in children with T1D is also influenced by several factors. A study from Iran investigating factors associated with insulin adherence among individuals with T1D showed that factors such as forgetting to buy insulin, physician inaccessibility, cost, exhaustion from long-term injections, forgetfulness, injection site reactions, and rebellion against parents were significantly associated with non-adherence to insulin therapy [7]. A cross-sectional study in Brazil examined factors associated with adherence to insulin therapeutic regimens. Adherence was positively associated with higher socioeconomic status, better glycemic control, and frequent self-monitoring of blood glucose. Barriers to adherence included cost, side effects, and difficulty in understanding the treatment plan [8]. Another study from Jordan showed that poor health literacy was associated with poor lower HRQoL in these patients [9].

Studies have shown that HRQoL can be improved with available interventions. A study from Spain which assessed HRQoL in children and adolescents with type 1 diabetes showed that older children and patients with poor metabolic control (HbA1c > 7.5%) had the lowest HRQoL [2]. A study from Saudi Arabia showed that older age, higher family socioeconomic status, excellent school performance, and higher parental education level, particularly in mothers, were significantly associated with higher total HRQoL score [10]. Yet another study from the UK evaluating metabolic outcomes and HRQoL of children with TID trained in the use of the Freestyle Flash Glucose Monitoring system found significant improvements in HbA1c and Pediatric Quality of Life Diabetes Module (PedsQL) 3.2 diabetes scores, indicating improved HRQoL and reduced diabetes symptoms and treatment barriers [11]. Therefore, it is crucial first to evaluate the HRQoL in children with T1D to determine the needs of these children and develop appropriate interventions. Furthermore, assessment of HRQoL can provide help understand into patients’ needs and preferences about the management of their illness in a setting like Bahrain where only a few studies have been previously conducted on HRQoL in patients with T1D.

Evaluating HRQoL in Bahrain, especially among children and adolescents with T1D, is critical due to the unique challenges posed by the region’s healthcare landscape and cultural context. Bahrain is one of the most affected countries by diabetes, with a diabetes mellitus incidence rate of 14.7% among its adult population. This high prevalence places a significant burden on the healthcare system and underscores the need for effective management and prevention strategies [12]. According to the International Diabetes Federation, the number of cases in Bahrain is projected to continue rising, mirroring trends across the Gulf Cooperation Council (GCC) countries. Predictions indicate that by 2030, approximately a quarter of the population in GCC countries will have diabetes mellitus, highlighting a regional public health crisis. In the Middle East and North Africa (MENA) region, the estimated percentage of new cases of type 1 diabetes in children aged 0–14 years was 18.1% in 2021. Specifically, within the Gulf Cooperation Council (GCC) countries, the rates were as follows: 2.7% in Saudi Arabia, 0.4% in Kuwait, 0.2% in Qatar, and 0.0% in both Oman and Bahrain. According to the International Diabetes Federation Atlas for Bahrain, demographic data show that the population of children aged 0–14 years was 226,000 in 2000, increasing to 315,300 in 2021 [13]. Studies have demonstrated that targeted interventions can significantly enhance HRQoL. In Bahrain, understanding the specific HRQoL issues faced by this population can guide the development of interventions tailored to their needs. The cultural and lifestyle factors in Bahrain, including dietary habits and family dynamics, may influence the management and outcomes of TID, making it essential to assess HRQoL within this specific context [1]. Moreover, recent studies have shown that online education and the use of technology, such as continuous glucose monitoring systems, have led to improvements in HRQoL and metabolic outcomes in children with TID. These findings suggest that similar interventions could be beneficial in Bahrain, but they need to be adapted to the local context to be truly effective. Additionally, measuring HRQoL can help identify gaps in the current healthcare services and provide insights into how healthcare providers can better support children with TID and their families. By understanding the factors that impact HRQoL, healthcare professionals can develop comprehensive, culturally appropriate care plans that address both medical and psychosocial needs [14]. Therefore, assessing HRQoL in Bahraini children with TID is crucial to identify their specific needs, improve their overall well-being, and inform the development of targeted interventions that can lead to better health outcomes and enhanced HRQoL.

To the best of our knowledge, no previous research has been conducted on HRQoL among children and adolescents with T1D in Bahrain. Our study aimed to assess the HRQoL of children and adolescents with T1D in Bahrain and to evaluate the associations between various factors and domains of HRQoL among children and adolescents with T1D in Bahrain.

## 2. Methods

### 2.1. Participants

In this cross-sectional study, 182 patients were enrolled from the diabetes outpatient clinic of the Salmaniya Medical Complex (Manama, Bahrain), between October 2023 and May 2024. Patients aged 5 to 16 years who had been diagnosed with T1D for at least six months were eligible for inclusion. These patients received treatment during regular follow-up checkups at the hospital’s diabetes clinic. Patients who were excluded were within six months of their initial diagnosis, were younger than 5 years or older than 16 years, or had other comorbid conditions (neurological or mental health conditions). Determining an appropriate sample size is crucial for ensuring the reliability and validity of study results. For this study, we aimed to achieve a confidence level of 95% and a margin of error of 5%. Using the formula for sample size calculation for proportions,

n = Z^2^ × *p*(1 − *p*)/E^2^

where

- n = required sample size;
- Z = Z-value (1.96 for a 95% confidence level);
- *p* = estimated proportion of the population (assumed to be 0.5 for maximum variability);
- E = margin of error (0.05).

This generated the required sample size of approximately 384 participants. However, considering practical constraints and the specific population of children with T1D in Bahrain, we adjusted our target sample size to 182 participants. This was because of the limited number of children and adolescents in the clinics. According to the hospital records, the total registered number of patients in the clinics at the time of the study was 710 adolescents and 290 children. Considering that these patients are also referred from the private sector as the cost of private care could be high, the hospital caters to almost 95% of patients with TID. This adjustment was based on the availability of eligible participants and ensured the feasibility of data collection within the study period while still providing sufficient power to detect significant associations and trends within this specific cohort.

### 2.2. Demographic and Diabetes-Related Information Questionnaire

The first part of the questionnaire collected demographic information, including age, gender, family income, school performance, weight, and height. Diabetes-related information included the mean age at diagnosis, duration of diabetes, type of insulin regimen, type of glucometer, number of emergency department visits in 2023, and mean HbA1c during previous visits.

### 2.3. Data Collection

Demographic data, including mean age at diagnosis, BMI, HbA1c, mean age at the start of using the Freestyle glucometer (manufactured by Abbott, Alameda, CA, USA), duration of diabetes, and insulin treatment regimen (subcutaneous/pump), were recorded. Face-to-face interviews were conducted during routine visits to the outpatient clinic of Salmaniya Medical Complex using the Arabic and English versions of the PedsQL 3.2 questionnaire [15].

### 2.4. Measures

The first part of the questionnaire consisted of socio-demographic information, including age, gender, family income, school performance, and weight and height. Diabetes-related information included age at diagnosis, duration of TID, type of insulin regimen, type of glucometer used, number of emergency department visits in the previous year, and mean HbA1c during the previous visits. The second part of the questionnaire included the PedsQL 3.2 Diabetes Module, a multi-dimensional, diabetes-specific instrument that assesses children, adolescents, and young adults with T1D. PedsQL is one of the most widely used measures to assess HRQoL in children [15]; we used its validated Arabic version [16]. This 23-item validated questionnaire evaluates a child’s health in terms of physical, emotional, social, and school functioning. It is commonly used in clinical trials to assess the effectiveness of medical treatments in children. The 32-item multidimensional instrument consists of four domains: diabetes symptoms (15 items), treatment barriers (5 items), treatment adherence (5 items), worry (3 items), and communication (4 items). A 5-point scale was used (0 = never, 1 = rarely, 2 = sometimes, 3 = very frequently, 4 = always). Items were reverse-scored and linearly transformed to a 0–100 scale, with higher scores indicating better HRQoL.

## 3. Data Analysis

All the collected information was analyzed using IBM SPSS Statistics 29.0 (IBM Corp. Released 2022. IBM SPSS Statistics for Windows, Version 29.0. Armonk, NY, USA: IBM Corp). For the descriptive purposes, categorical information was presented using frequency and percentages, and continuous information was presented using mean with standard deviation (SD) or median with interquartile. Normally distributed diabetes symptom mean scores were compared with categorical associations using the independent *t*-test or the ANOVA test. Other not normally distributed domain scores were tested using Mann–Whitney or Kruskal–Wallis tests. Significant factors and non-significant factors up to 0.2 levels were taken into generalized linear regression model for adjusting the confounding factors. A *p*-value of <0.05 was considered as statistical significance.

## 4. Results

A total of 182 patients were enrolled in this study. Table 1 presents the demographic and clinical characteristics; the mean age of the participants was 12.21 years with a standard deviation of 2.86 years. The majority (50.0%) fell within the age group of 13–16 years, followed by 9–12 years (36.8%) and 6–8 years (13.2%). The mean age at diagnosis was 6.83 years (SD = 3.11), with the largest proportion (57.7%) diagnosed between 6 and 11 years. The sample is fairly balanced in terms of gender, with 52.2% males and 47.8% females. The mean BMI was 21.89 (SD = 6.90), with most patients (47.8%) having a BMI between 18.5 and 24.99. Regarding family income, the majority (49.5%) fell in the 500–1000 income category. Most patients attended secondary school (71.4%) and reported excellent school performance (58.2%). Three-fourths of patients had an HbA1c level greater than 7.5%. The majority (62.6%) of patients used a standard glucose meter. Insulin therapy was predominantly subcutaneous (45.6%). A few patients (9.3%) reported chronic disease or mental illness. Lastly, a majority (53.8%) had no emergency department visits, and 60.4% had a diabetic ketoacidosis episode in 2023.

**Figure 1:**
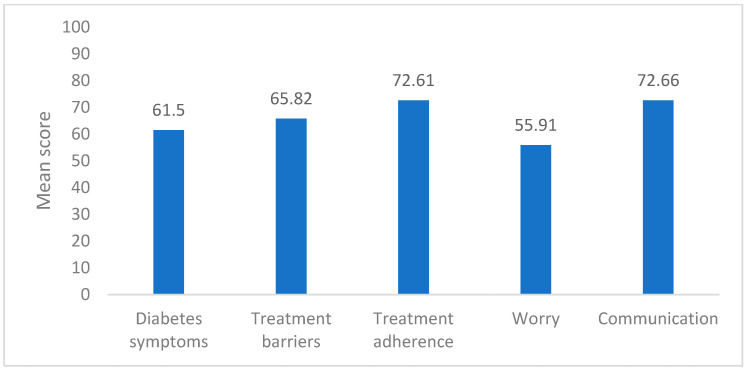
Distribution of various domain mean scores (n = 182).

Table 2 provides the distribution of domain scores related to diabetes management among the study participants. The domain scores are summarized with their mean values and standard deviations (SD) (Figure 1), as well as median values with interquartile ranges (IQR) and minimum and maximum scores. Treatment adherence had the highest median score of 80.0 (60.0–91.25). Diabetes symptoms were reported as the mean score of 61.50 ± 14.10 and communication domains were reported with the median of 75.0 (56.25–93.75). The worry domain had the lowest median score at 58.33 (39.58–75.0).

Table 3 shows the associations between demographic and other characteristics with mean diabetes symptom scores, median scores of treatment barriers, and treatment adherence and *p*-values. In regard to diabetes mean score, age groups showed the mean symptom scores of 64.31 ± 13.25, 61.54 ± 14.57, and 60.73 ± 14.03, respectively, with a non-significant *p*-value of 0.546. Family income showed a significant association (*p* = 0.009), with mean scores ranging from 56.97 ± 14.26 to 64.54 ± 14.21 across income categories. A child’s school performance also showed highly significant association (*p* < 0.001) with mean scores ranging from 41.33 ± 16.35 for poor performance to 64.43 ± 14.04 for excellent performance. Age at diagnosis, sex, BMI, school, type of glucometer, insulin regimen, presence of chronic illness, number of emergency department visits, and number of diabetic ketoacidosis during in 2023 did not show any statistically significant associations with the diabetes symptoms. The multivariate analysis (Table 4) showed several significant associations with diabetes symptom scores. Participants from families with incomes between 500 and 1000 showed a positive β-coefficient (β = 4.69, *p* = 0.032), poor school performance significantly correlated with lower symptom scores (β = −22.986, *p* < 0.001), and lower HbA1c reported positive symptoms (β = 6.55, *p* = 0.003). No diabetic ketoacidosis reported higher symptoms (β = 10.13, *p* = 0.032).

In regard to the treatment barriers (Table 3), age group showed a significant association (*p* < 0.001), with median scores increasing from 57.50 (IQR 45.0–73.75) for ages 6–8 to 70.0 (IQR 60.0–85.0) for ages 13–16. School type also showed a significant association (*p* = 0.001), where primary school students report a lower median score of 57.50 (IQR 45.0–73.75) compared to secondary school students with a median score of 70.0 (IQR 60.0–80.0). Additionally, the type of insulin regimen used significantly affected treatment barriers (*p* < 0.001), with patients on pump therapy reporting higher median scores (75.0, IQR 60.0–85.0) than those on subcutaneous insulin (60.0, IQR 45.0–70.0). Other variables such as age at diagnosis, sex, BMI, family income, child school performance, HbA1c level, presence of chronic disease or mental illness, number of emergency department visits, and diabetic ketoacidosis did not show statistically significant associations with treatment barriers. In the multivariate analysis (Table 5), age group 9–12 years demonstrated a negative association with scores (β = −10.041, *p* = 0.004), the standard glucometer showed less barriers compared to continuous (β = −5.056, *p* = 0.006), and the type of insulin regimen played a role with subcutaneous administration negatively impacting barriers (β = −10.076, *p* = 0.001) compared to pump usage.

In association with the treatment adherence, age group showed a significant association with treatment adherence (*p* < 0.001), with adherence scores from 50.0 (35.0–70.0) in the 6–8 age group to 85.0 (70.0–100.0) in the 13–16 age group. Similarly, age at diagnosis also showed statistical significance (*p* < 0.001), from 70.0 (50.0–83.33) for 1–2 years, 65.0 (36.25–88.75) for 2–5, 80.0 (65.0–95.0) for 6–11, and 95.0 (82.5–100.0) for 12–16 years. Males showed higher at 85.0 (60.0–95.0) compared to females at 70.0 (55.0–85.0) and was significant (*p* = 0.013). BMI categories showed statistically significant differences (*p* = 0.036); individuals with a BMI < 18.5 had a median score of 70.0 (83.75–91.25), BMI of 18.5–24.99 had a median of 80.0 (65.0–5.0), BMI of 25–29.99 had a median score of 80.0 (41.25–90.0), and those with a BMI ≥ 30 had a median score of 60.0 (IQR 25.0 to 90.0). Primary school students reported a lower median score of 62.50 (45.0, 85.0) compared to secondary school students with a median score of 80.0 (65.0, 95.0), which was significant (*p* < 0.001). Those who had a mental illness showed a lower adherence median score that was statistically significant. All other variables did not show any statistically significant difference. In the multivariate analysis, younger age groups (6–8 and 9–12 years) exhibited negative associations with scores (β = −20.651 and β = −12.002, respectively, both *p* < 0.01), indicating poorer adherence compared to older adolescents. BMI categories (<18.5, 18.5–24.99, 25–29.99) also showed positive associations with adherence scores (all *p* < 0.05). Having a chronic disease or mental illness negatively impacted adherence (β = −12.286, *p* = 0.021).

Table 4 shows the associations between demographic and other characteristics with median worry domain and communications domain scores and *p*-values. Age group showed no significant associations with worry scores (*p* = 0.712). Age at diagnosis exhibited a borderline significant trend (*p* = 0.064), where the older age group of 12–16 years reported higher worry scores with a median of 75.0 (50.0–93.75). Sex was significantly associated with worry (*p* = 0.002), with males reporting higher median scores at 66.67 (50.0–83.33) compared to females at 50.0 (33.33–66.67). Child’s school performance showed significant association (*p* = 0.029); poorly performing children reported a higher worry score at 100 (79.17–100.0), and HbA1c levels (*p* = 0.019) demonstrated significant associations with the median scores. Other variables such as BMI, family income, school, type of glucometer, insulin regimen, number of emergency department visits, and diabetic ketoacidosis in 2023 did not show statistically significant associations (*p* > 0.05). In the multivariate analysis (Table 5), males showed a significant positive association (β = 12.113, *p* = 0.003), indicating higher worry scores compared to females. Family income and school performance did not show statistically significant associations with worry scores. Children with poor school performance showed higher positive association (β = 32.180, *p* = 0.010).

Age group exhibited a statistically significant association with communication scores (*p* = 0.005), where older age groups aged 13–16 years recorded a higher median score of 75.0 (62.50–100.0) compared to younger groups aged 6–8 years at 71.88 (39.06–85.94). Age at diagnosis also showed a highly significant association (*p* < 0.001), with communication scores increasing significantly with older age at diagnosis, 6–11 years at 81.25 (62.50–100.0) and 12–16 years at 90.63 (67.19–100.0). School type showed a significant association (*p* = 0.045), where students at secondary school at 75.0 (56.25–100.0) reported higher communication scores compared to those at primary school at 75.0 (45.31, 87.50). The other variables of sex, BMI, family income, child’s school performance, HbA1c levels, type of glucometer, insulin regimen, presence of chronic disease or mental illness, number of emergency department visits, and number of diabetic ketoacidosis episodes did not show statistically significant associations with communication scores (*p* > 0.05). In the multivariate analysis (Table 5), younger age groups (6–8 and 9–12 years) showed negative associations with scores (both *p* < 0.05), and the type of insulin regimen also influenced communication scores, with subcutaneous administration positively impacting communication (β = 7.737, *p* = 0.024) compared to pump usage.

## 5. Discussion

The current study assessed the HRQoL of 182 Bahraini children and adolescents with T1D using the PedsQL 3.2 scale. The mean HRQoL score was 65.7 ± 22.51, suggesting an average level of HRQoL among this population. Several significant associations between various factors and diabetes symptom scores, as well as other HRQoL domains, were noted in this study. Notably, participants from families with incomes between 500 and 1000 Bahraini Dinar exhibited better diabetes symptom scores, suggesting that higher family income may provide better access to healthcare resources and support systems. Poor school performance was significantly correlated with lower diabetes symptom scores, highlighting the impact of academic challenges on diabetes management. Additionally, lower HbA1c levels were positively associated with better diabetes symptom scores, underscoring the importance of maintaining good glycemic control. The absence of diabetic ketoacidosis episodes was independently associated with higher diabetes symptom scores, indicating that preventing such episodes can significantly improve the HRQoL for these patients.

Our findings on HRQoL scores were comparable with a regional study that showed a guardian-proxy mean of the PedsQoL-DM score of 64.7% [7]. Despite variations in mean PedsQL-DM scores among studies, most studies, including this one, demonstrated a comparable range of HRQoL (60.5–80.3%). A study from Japan reported a higher HRQoL in children and adolescents with TID compared to type 2 diabetes mellitus [17]. Our findings are also consistent with a cross-sectional study from Saudi Arabia that assessed HRQoL among children and adolescents with TID, finding a mean total score of 72.61 ± 15.36. A similar cross-sectional study in India found a mean total PedsQL Diabetes Module self-report score of 83.77 ± 11.11 for 5–12-year-olds and 80.27 ± 13.52 for 13–18-year-olds. Factors positively associated with HRQoL included attending education, educated mothers, and insulin injections by primary caregivers [14].

Factors associated with a low HRQoL included age, longer TID duration, and insulin administration three times per day. Factors associated with higher HRQoL included older age, higher socioeconomic status, excellent school performance, and higher parental education in our study [18]. Conversely, educated parents, employed fathers, and recurrent blood glucose checking were associated with higher HRQoL [19]. A cross-sectional study in Saudi Arabia found that the mean HRQoL was 0.71 +/− 0.21, indicating a relatively moderate quality of life. Gender, marital status, age, education, employment, and type of medication positively influenced participants’ HRQoL [20].

Treatment adherence had the highest median score (72.61), suggesting that most participants adhered moderately well to their treatment regimens. However, there was significant variation, with younger age groups and those with chronic illnesses showing lower adherence. This is similar to findings from studies from Saudi Arabia [20,21] and Iran [7]. However, not all studies corroborate with our findings. For instance, a study from Brazil showed higher insulin adherence among older age groups. This could be explained by several factors identified in studies on adherence in children with T1D. Firstly, younger children often lack the developmental skills necessary for independent management, requiring significant parental support. Secondly, the fear of injections and the disruption of daily routines can lead to resistance in younger patients. Thirdly, children with chronic illnesses, like TID, may experience treatment fatigue over time, making adherence more challenging [14].

Communication domain scores were relatively high (median: 72.66) in our study, indicating open communication between children and caregivers regarding diabetes management. However, younger children and those using insulin pumps communicated less effectively. This finding is consistent with research on developmental stages, where younger children may lack the necessary communication skills. Interestingly, a study [22] exploring communication between patients and healthcare providers using insulin pumps suggests a potential explanation for the lower communication scores in pump users. The study found that some patients with pumps felt their healthcare providers lacked adequate training on pump therapy. This could lead to a communication gap between patients and caregivers, with children on pumps feeling less supported or having unanswered questions about their specific management. In contrast, on the general impact of insulin pumps on HRQoL, it suggests pumps may empower patients and improve their sense of self-efficacy in managing their diabetes [23]. This seemingly contradictory finding highlights the need for further investigation into how pump therapy specifically affects communication dynamics within families.

The worry domain had the lowest score (median: 55.91), highlighting potential anxieties related to managing TID. This finding suggests that while participants might generally cope well with the disease, anxieties around specific aspects of TID management persist. Notably, males and children with poorer school performance reported higher worry scores. This could be due to societal expectations around masculinity leading to under-expression of worries in boys [24]. Furthermore, the additional burden of managing TID could be impacting focus and concentration at school, leading to higher worry scores in children with lower academic performance [25,26].

Scores were moderate for diabetes-related symptoms (median: 61.50), suggesting participants experienced some diabetes-related symptoms. However, factors like poor glycemic control, lower family income, and a higher number of emergency department visits were associated with worse symptom scores. This is comparable with findings from other studies on children with TID, highlighting the presence of these symptoms despite generally good HRQoL [27]. Poor glycemic control, as expected, played a significant role. This is supported by research demonstrating an associated between uncontrolled blood sugar levels and increased physical symptoms [4]. Additionally, frequent emergency department visits might indicate a more complex disease course or challenges with managing symptoms at home [28,29].

Our findings showed that family income played a crucial role in the management and HRQoL for children with T1D. Lower family income and a higher number of emergency department visits were factors associated with worse symptom scores. This suggests a potential link between socioeconomic status and symptom management. Socioeconomic factors can influence access to quality healthcare, healthy food options, and stress management resources, all of which can impact how children experience and manage their symptoms. Higher family income was associated with better diabetes symptom scores, indicating that families with more financial resources can afford better healthcare, access to advanced diabetes management tools, and a more supportive environment. This financial stability allows for consistent medical follow-ups, access to continuous glucose monitoring systems, and the ability to maintain a healthy diet, all of which contribute to improved HRQoL for children with T1D. Studies have shown that socioeconomic status significantly impacts glycemic control and adherence to treatment, further emphasizing the importance of financial resources in managing chronic conditions like T1D [30]. Additionally, families with higher incomes are more likely to afford educational resources and extracurricular activities that can enhance the overall well-being of children with T1D [31].

Maintaining lower HbA1c levels is critical for improving the HRQoL in patients with T1D, as shown in our study. HbA1c is a measure of average blood glucose levels over the past two to three months, and lower levels indicate better glycemic control [32]. Good glycemic control is associated with fewer diabetes-related complications, better physical health, and overall improved HRQoL. Studies have demonstrated that children with lower HbA1c levels experience fewer symptoms of diabetes, have better school performance, and engage more in social activities [33]. Effective management strategies, including regular monitoring and adherence to insulin therapy, are essential for maintaining optimal HbA1c levels and enhancing the well-being of children with T1D. Furthermore, lower HbA1c levels are linked to reduced risks of long-term complications, such as cardiovascular diseases and neuropathy, which can significantly impact the HRQoL by reducing stress in patients with diabetes [34].

School performance was another significant factor identified in our study affecting the HRQoL of children with T1D. Poor academic performance is correlated with lower diabetes symptom scores, indicating that children struggling academically may also face challenges in managing their diabetes. The stress and anxiety associated with poor school performance can exacerbate diabetes symptoms and negatively impact overall well-being [35]. Conversely, children who perform well academically tend to have better diabetes management and higher HRQoL. Most probably it is because these students receive better health education and medical support while in school, as suggested by a regional study [21]. This also highlights the need for integrated support systems that address both educational and health needs, ensuring that children with T1D receive the necessary resources and assistance to in school and manage their condition effectively. Additionally, schools contributed by facilitation and support for students with T1D, such as flexible schedules for blood glucose monitoring and insulin administration.

Scores were moderate (median: 65.82), indicating some challenges in managing diabetes treatment. This finding aligns with research highlighting the complexities of adherence in children with TID. Importantly, several factors influenced these barriers. As expected, younger children face greater difficulties due to developmental limitations and socioeconomic status [14]. The impact of specific diabetes technology on treatment barriers appears to be less clear-cut. While some studies have not found a significant difference in adherence based on glucometer features, others suggest that newer, more user-friendly models might improve adherence. Similarly, the influence of insulin regimens is complex. While complex regimens might pose challenges, appropriate pump therapy can improve adherence in some cases [36].

In Bahrain, there is governmental support alongside charitable organizations that provide insulin pumps for patients. Additionally, a specialized clinic for insulin pump education is conducted every Monday at the SMC. From our observations, significant resources are invested in educating parents about the children with T1D. The diabetic clinic at SMC conducts various activities, including diabetes camps for both patients and their families. Most schools in Bahrain have a nurse, and it would be beneficial to conduct regular educational sessions for them, helping to guide how to manage diabetes-related emergencies effectively. Psychological support is essential for individuals diagnosed with diabetes, as it is a lifelong condition that requires continuous management. Regular psychological sessions are recommended help children and their families cope with the emotional and mental challenges of living with T1D, including managing the stress of constant monitoring and lifestyle adjustments.

## 6. Strengths and Limitations

This study assessing the HRQoL of children and adolescents with T1D in Bahrain has several strengths and weaknesses. A key strength is its focus on an understudied population in Bahrain, addressing a gap in existing literature regarding HRQoL in children with T1D in this specific cultural context. The use of the validated PedsQL 3.2 Diabetes Module, along with the collection of demographic and diabetes-related data, allowed for a comprehensive assessment of HRQoL and the exploration of potential associated factors. However, the study also has limitations. The sample size (n = 182) for this special group of the population in Bahrain was smaller than originally estimated. This is because of the patient availability, despite the fact that the hospital is the major tertiary care hospital of the country attending to the most patients with T1D. As the country has a small overall population, most patients with T1D attend these clinics where the total registered load of adolescent and child patients is about 1000 at any time. Therefore, although a smaller sample size is a limitation, the sample size is sufficient considering the patient population size. This is alongside the fact that cross-sectional design limits the ability to draw causal inferences between the identified factors and HRQoL.

## 7. Conclusions

This study’s findings have significant implications for T1D management in children and adolescents. The associations between HRQoL and factors like family income, school performance, HbA1c levels, and ketoacidosis episodes identifies the need for interventions addressing both medical and psychosocial aspects of care provided to these patients in Bahrain. Specifically, socioeconomic support and health education may improve diabetes symptom scores. Effective glycemic control and complication prevention are also the core components of improving the quality of life of these patients. Younger children require support for treatment adherence, given the negative impact of age on treatment barriers. Device and treatment method preferences (glucometer type, insulin administration) should be considered to minimize treatment burden and improve communication. Finally, gender-specific differences in worry and the impact of comorbidities on adherence required patient centered plans for the overall well-being and HRQoL in this population.

## Figures and Tables

**Table 1:** Distribution of patient demographic and other characteristics (n = 182).

Variables	n	%
**Age (mean ± SD)**	12.21 ± 2.86	
**Age group**		
6–8	24	13.2
9–12	67	36.8
13–16	91	50.0
**Age at diagnosis (mean ± SD)**	6.83 ± 3.11	
**Age at diagnosis**		
1–2 years	15	8.2
2–5 years	48	26.4
6–11 years	105	57.7
12–16 years	14	7.7
**Sex**		
Male	95	52.2
Female	87	47.8
**BMI (mean ± SD)**	21.89 ± 6.90	
**BMI**		
<18.5	58	31.9
18.5–24.99	89	48.9
25–29.99	20	11.0
≥30	15	8.2
**Family’s income**		
<500	44	24.2
500–1000	90	49.5
>1000	48	26.4
**School**		
Primary	52	28.6
Secondary	130	71.4
**Child’s school performance**		
Poor	5	2.7
Good	24	13.2
Very good	47	25.8
Excellent	106	58.2
**HbA1c group**		
≤7.5	44	24.2
>7.5	138	75.8
**Type of glucometer**		
Standard glucose meter	114	62.6
Continuous glucose monitor	68	37.4
**What type of insulin regimens is the patient in?**		
Subcutaneous	83	45.6
Pump	99	54.4
**Do you have any chronic disease or mental illness?**		
Yes	17	9.3
No	165	90.7
**Number of emergency department visits in 2023**		
0	98	53.8
1–2	65	35.7
>2	19	10.4
**Number of diabetic ketoacidosis in 2023**		
0	110	60.4
1–2	58	31.9
>2	14	7.7

**Table 2:** Distribution of domain scores (n = 182).

Domain	Mean ± SD	Median (IQR)	Minimum	Maximum
Diabetes symptoms	61.50 ± 14.10	61.67 (51.67, 71.67)	20.0	98.33
Treatment barriers	65.82 ± 19.89	67.50 (50.0, 80.0)	20.0	100.0
Treatment adherence	72.61 ± 23.92	80.0 (60.0, 91.25)	5.0	100.0
Worry	55.91 ± 28.85	58.33 (39.58, 75.0)	0	100.0
Communication	72.66 ± 23.11	75.0 (56.25, 93.75)	6.25	100.0

**Table 3:** Association between demographics and other characteristics with mean diabetes symptoms, median treatment barriers, and treatment adherence domain scores (n = 182).

Variables	Diabetes Symptoms Mean ± SD	*p*-Value	Treatment Barriers Median (IQR)	*p*-Value	Treatment Adherence Median (IQR)	*p*-Value
**Age group**						
6–8	64.31 ± 13.25	0.546 *	57.50 (45.0, 73.75)	<0.001 ^#^	50.0 (35.0, 70.0)	<0.001 ^#^
9–12	61.54 ± 14.57		65.0 (50.0, 70.0)		70.0 (55.0, 85.0)	
13–16	60.73 ± 14.03		70.0 (60.0, 85.0)		85.0 (70.0, 100.0)	
**Age at diagnosis**						
1–2 years	58.44 ± 18.12	0.759 *	60.0 (45.0, 85.0)	0.655 ^#^	70.0 (50.0, 83.33)	<0.001 ^#^
2–5 years	60.80 ± 14.13		67.50 (45.0, 80.0)		65.0 (36.25, 88.75)	
6–11 years	62.32 ± 13.66		70.0 (55.0, 80.0)		80.0 (65.0, 95.0)	
12–16 years	61.07 ± 13.50		67.50 (60.0, 90.0)		95.0 (82.5, 100.0)	
**Sex**						
Male	61.54 ± 13.83	0.967 ^	65.0 (50.0, 80.0)	0.508 ^@^	85.0 (60.0, 95.0)	0.013 ^@^
Female	61.46 ± 14.48		70.0 (50.0, 80.0)		70.0 (55.0, 85.0)	
**BMI**						
<18.5	59.28 ± 14.71	0.547 *	65.0 (48.75, 75.0)	0.398 ^#^	70.0 (83.75, 91.25)	0.036 ^#^
18.5–24.99	62.40 ± 13.62		70.0 (57.50, 80.0)		80.0 (65.0, 95.0)	
25–29.99	62.92 ± 14.20		70.0 (51.25, 80.0)		80.0 (41.25, 90.0)	
≥30	62.89 ± 14.86		60.0 (45.0, 85.0)		60.0 (25.0, 90.0)	
**Family’s income**						
<500	56.97 ± 14.26	0.009 *	65.0 (40.0, 75.0)	0.076 ^#^	85.0 (45.0, 100.0)	0.071 ^#^
500–1000	64.54 ± 14.21		70.0 (50.0, 80.0)		80.0 (63.75, 91.25)	
>1000	59.97 ± 12.58		70.0 (56.25, 80.0)		70.0 (60.0, 80.0)	
**School**						
Primary	61.70 ± 14.53	0.906 ^	57.50 (45.0, 73.75)	0.001 ^@^	62.50 (45.0, 85.0)	<0.001 ^@^
Secondary	61.42 ± 13.99		70.0 (60.0, 80.0)		80.0 (65.0, 95.0)	
**Child’s school performance**						
Poor	41.33 ± 16.35	<0.001 *	60.0 (52.50, 72.50)	0.787 ^#^	70.0 (35.0, 92.50)	0.905 ^#^
Good	58.89 ± 9.16		67.50 (50.0, 80.0)		75.0 (55.0, 93.75)	
Very good	58.37 ± 13.79		65.0 (45.0, 75.0)		80.0 (55.0, 95.0)	
Excellent	64.43 ± 14.04		70.0 (50.0, 80.0)		80.0 (60.0, 91.25)	
**HbA1c group**						
≤7.5	67.31 ± 14.69	0.002 ^	70.0 (60.0, 78.75)	0.412 ^@^	70.0 (52.50, 93.75)	0.439 ^@^
>7.5	59.64 ± 13.45		65.0 (48.75, 80.0)		80.0 (60.0, 91.25)	
**Type of glucometer**						
Standard	62.31 ± 14.12	0.318 ^	65.0 (45.0, 75.0)	0.029 ^@^	80.0 (58.75, 90.0)	0.163 ^@^
Continuous	60.15 ± 14.07		70.0 (60.0, 80.0)		80.0 (60.0, 100.0)	
**What type of insulin regimens is the patient in?**						
Subcutaneous	62.29 ± 14.11	0.492 ^	60.0 (45.0, 70.0)	<0.001	80.0 (50.0, 95.0)	0.815 ^@^
Pump	60.84 ± 14.14		75.0 (60.0, 85.0)		80.0 (60.0, 90.0)	
**Do you have any chronic disease or mental illness?**						
Yes	62.35 ± 14.39	0.795 ^	70.0 (45.0, 77.50)	0.718 ^@^	60.0 (42.50, 80.0)	0.008 ^@^
No	61.41 ± 14.12		65.0 (50.0, 80.0)		80.0 (60.0, 95.0)	
**Number of EMD visits in 2023**						
0	65.39 ± 12.71	<0.001	70.0 (58.75, 85.0)	0.516 ^#^	80.0 (60.0, 95.0)	0.656 ^#^
1–2	57.82 ± 15.43		65.0 (45.0, 75.0)		80.0 (55.0, 90.0)	
>2	54.04 ± 9.66		65.0 (45.0, 75.0)		80.0 (60.0, 95.0)	
**Number of diabetic ketoacidosis in 2023**						
0	65.24 ± 13.60	<0.001 *	70.0 (53.75, 85.0)	0.647 ^#^	80.0 (60.0, 95.0)	0.689 ^#^
1–2	56.55 ± 13.60		65.0 (50.0, 75.0)		80.0 (60.0, 90.0)	
>2	52.62 ± 9.71		62.50 (45.0, 85.0)		72.5 (53.75, 95.0)	

^ Independent *t*-test, * ANOVA test, ^@^ Mann–Whitney ‘U’, ^#^ Kruskal–Wallis test.

**Table 4:** Association between demographics and other characteristics with worry and communication domain median scores (n = 182).

Variables	Worry Median (IQR)	*p*-Value	Communication Median (IQR)	*p*-Value
**Age group**				
6–8	50.0 (18.75, 93.75)	0.712 ^#^	71.88 (39.06, 85.94)	0.005 ^#^
9–12	58.33 (41.67, 75.0)		75.0 (50.0, 87.50)	
13–16	58.33 (33.33, 75.0)		75.0 (62.50, 100.0)	
**Age at diagnosis**				
1–2 years	50.0 (25.0, 83.33)	0.064 ^#^	62.5 (37.5, 75.0)	<0.001 ^#^
2–5 years	50.0 (25.0, 66.67)		62.50 (45.31, 75.0)	
6–11 years	58.33 (41.67, 75.0)		81.25 (62.50, 100.0)	
12–16 years	75.0 (50.0, 93.75)		90.63 (67.19, 100.0)	
**Sex**				
Male	66.67 (50.0, 83.33)	0.002 ^@^	75.0 (62.50, 93.75)	0.408 ^@^
Female	50.0 (33.33, 66.67)		75.0 (50.0, 93.75)	
**BMI**				
<18.5	58.33 (41.67, 75.0)	0.691 ^#^	75.0 (50.0, 89.06)	0.313 ^#^
18.5–24.99	58.33 (41.67, 75.0)		75.0 (59.38, 100.0)	
25–29.99	50.0 (12.5, 83.33)		75.0 (40.63, 96.88)	
≥30	66.67 (33.33, 100.0)		75.0 (56.25, 87.50)	
**Family’s income**				
<500	50.0 (16.67, 75.0)	0.075 ^#^	75.0 (57.81, 100.0)	0.552 ^#^
500–1000	58.33 (41.67, 83.33)		75.0 (54.69, 100.0)	
>1000	50.0 (33.33, 66.67)		75.0 (50.0, 87.50)	
**School**				
Primary	58.33 (41.67, 75.0)	0.883 ^@^	75.0 (45.31, 87.50)	0.045 ^@^
Secondary	58.33 (33.33, 75.0)		75.0 (56.25, 100.0)	
**Child’s school performance**				
Poor	100.0 (79.17, 100.0)	0.029 ^#^	62.50 (40.63, 81.25)	0.268 ^#^
Good	50.0 (27.08, 75.0)		87.50 (70.31, 100.0)	
Very good	58.33 (41.67, 75.0)		75.0 (50.0, 100.0)	
Excellent	58.33 (33.33, 77.08)		75.0 (50.0, 89.06)	
**HbA1c group**				
≤7.5	66.67 (41.67, 97.92)	0.019 ^@^	75.0 (51.56, 92.19)	0.540 ^@^
>7.5	50.0 (33.33, 75.0)		75.0 (56.25, 100.0)	
**Type of glucometer**				
Standard	50.0 (33.33, 75.0)	0.219 ^@^	75.0 (56.25, 100.0)	0.317 ^@^
Continuous	58.33 (41.67, 81.25)		75.0 (51.56, 87.50)	
**What type of insulin regimens is the patient in?**				
Subcutaneous	58.33 (41.67, 83.33)	0.201 ^@^	75.0 (62.50, 100.0)	0.164 ^@^
Pump	50.0 (33.33, 75.0)		75.0 (50.0, 93.75)	
**Do you have any chronic disease or mental illness?**				
Yes	50.0 (25.0, 75.0)	0.315 ^@^	75.0 (53.13, 90.63)	0.901 ^@^
No	58.33 (41.67, 75.0)		75.0 (56.25, 93.75)	
**Number of emergency department visits in 2023**				
0	50.0 (33.33, 66.67)	0.357 ^#^	75.0 (50.0, 100.0)	0.949 ^#^
1–2	58.33 (33.33, 83.33)		75.0 (56.25, 93.75)	
>2	66.67 (41.67, 100.0)		75.0 (43.75, 93.75)	
**Number of diabetic ketoacidosis in 2023**				
0	54.17 (41.67, 75.0)	0.710 ^#^	75.0 (50.0, 100.0)	0.646 ^#^
1–2	62.50 (33.33, 83.33)		75.0 (56.25, 89.06)	
>2	54.17 (33.33, 75.0)		81.25 (59.38, 93.75)	

^@^ Mann–Whitney ‘U’, ^#^ Kruskal–Wallis test.

**Table 5:** Multivariate analysis showing the association of quality of life domains with sociodemographic characteristics (n = 182).

Domain	Variables	β-Coefficient	95% C.I.		*p*-Value ^§^
**Diabetic symptoms**	**Family income**				
	<500	0.778	−4.355	5.910	0.767
	500–1000	4.69	0.392	8.987	0.032
	>1000	1.0			
	**Child’s school performance**				
	Poor	−22.986	−34.176	−11.796	<0.001
	Good	−0.487	−6.133	5.158	0.866
	Very good	−4.569	−8.786	−0.353	0.034
	Excellent	1.0			
	**HbA1c group**				
	≤7.5	6.551	2.202	10.899	0.003
	>7.5	1.0			
	**Number of emergency department visits in 2023**				
	0	2.284	−6.035	10.603	0.591
	1–2	1.131	−6.775	9.038	0.779
	>2	1.0			
	**Number of diabetic ketoacidoses in 2023**				
	0	10.125	0.877	19.373	0.032
	1–2	2.954	−5.973	11.881	0.517
	>2	1.0			
**Treatment barriers**	**Age group**				
	6–8	−7.609	−19.495	4.276	0.210
	9–12	−10.041	−16.819	−3.262	0.004
	13–16	1.0			
	**Family income**				
	<500	−4.546	−12.115	3.023	0.239
	500–1000	2.643	−3.841	9.126	0.424
	>1000	1.0			
	**School**				
	Primary	−1.141	−9.855	7.573	0.798
	Secondary	1.0			
	**Type of glucometer**				
	Standard	−5.056	−10.775	0.664	0.006
	Continuous	1.0			
	**What type of insulin regimens is the patient in?**				
	Subcutaneous	−10.076	−15.877	−4.276	0.001
	Pump	1.0			
**Treatment adherence**	**Age group**				
	6–8	−20.651	−35.101	−6.200	0.005
	9–12	−12.002	−20.074	−3.930	0.004
	13–16	1.0			
	**Age at diagnosis**				
	1–2 years	−12.809	−28.182	2.564	0.102
	2–5 years	−13.091	−26.264	0.081	0.051
	6–11 years	−4.526	−16.452	7.399	0.457
	12–16 years	1.0			
	**Sex**				
	Male	4.933	−1.340	11.206	0.123
	Female	1.0			
	**BMI group**				
	<18.5	17.252	5.331	29.173	0.005
	18.5–24.99	16.112	4.656	27.569	0.006
	25–29.99	14.473	1.006	27.941	0.035
	≥30	1.0			
	**Family income**				
	<500	7.583	−1.001	16.167	0.083
	500–1000	6.679	−0.651	14.010	0.074
	>1000	1.0			
	**School**				
	Primary	−0.079	−10.625	10.467	0.988
	Secondary	1.0			
	**Type of glucometer**				
	Standard	−9.443	−15.841	−3.045	0.004
	Continuous	1.0			
	**Do you have any chronic disease or mental illness**				
	Yes	−12.286	−22.735	−1.837	0.021
	No	1.0			
**Worry**	**Age at diagnosis**				
	1–2 years	−15.102	−34.763	4.560	0.132
	2–5 years	−16.033	−32.641	0.575	0.058
	6–11 years	−11.027	−26.334	4.279	0.158
	12–16 years	1.0			
	**Sex**				
	Male	12.113	4.062	20.164	0.003
	Female	1.0			
	**Family income**				
	<500	−1.628	−12.768	9.513	0.775
	500–1000	7.822	−1.597	17.240	0.104
	>1000	1.0			
	**Child’s school performance**				
	Poor	32.180	7.789	56.570	0.010
	Good	−10.213	−22.598	2.173	0.106
	Very good	−3.820	−13.329	5.689	0.431
	Excellent	1.0			
	**HbA1c group**				
	≤7.5	5.233	−4.197	14.663	0.277
	>7.5	1.0			
**Communication**	**Age group**				
	6–8	−16.325	−30.973	−1.677	0.029
	9–12	−9.305	−17.585	−1.024	0.028
	13–16	1.0			
	**School**				
	Primary	−1.275	−12.094	9.543	0.817
	Secondary	1.0			
	**What type of insulin regimens is the patient in?**				
	Subcutaneous	7.737	1.018	14.457	0.024
	Pump	1.0			

^§^ Generalized linear model.

## Data Availability

The raw data supporting the conclusions of this article will be made available by the authors upon request.

## References

[1] Hilliard M.E., Minard C.G., Marrero D.G., de Wit M., Thompson D., DuBose S.N., Verdejo A., Monzavi R., Wadwa R.P., Jaser S.S. (2020). Assessing Health-Related Quality of Life in Children and Adolescents with Diabetes: Development and Psychometrics of the Type 1 Diabetes and Life (T1DAL) Measures. J. Pediatr. Psychol..

[2] Murillo M., Bel J., Perez J., Corripio R., Carreras G., Herrero X., Mengibar J.M., Rodriguez-Arjona D., Ravens-Sieberer U., Raat H. (2017). Health-related quality of life (HRQOL) and its associated factors in children with Type 1 Diabetes Mellitus (T1DM). BMC Pediatr..

[3] Bekele B.T., Demie T.G., Worku F. (2022). Health-Related Quality-of-Life and Associated Factors Among Children and Adolescents with Type 1 Diabetes Mellitus: A Cross-Sectional Study. Pediatric. Health Med. Ther..

[4] Streisand R., Monaghan M. (2014). Young children with type 1 diabetes: Challenges, research, and future directions. Curr. Diab. Rep..

[5] Al-Agha A.E., Alafif M.M., Abd-Elhameed I.A. (2015). Glycemic control, complications, and associated autoimmune diseases in children and adolescents with type 1 diabetes in Jeddah, Saudi Arabia. Saudi Med. J..

[6] Ausili E., Tabacco F., Focarelli B., Padua L., Crea F., Caliandro P., Pazzaglia C., Marietti G., Rendeli C. (2007). Multidimensional study on quality of life in children with type 1 diabetes. Eur. Rev. Med. Pharmacol. Sci..

[7] Fayyaz F., Aghamahdi F., Noorian S., Tabatabaei-Malazy O., Qorbani M. (2022). Associated factors to insulin adherence in type 1 diabetes in Tehran and Karaj, Iran. J. Diabetes Metab. Disord..

[8] Gomes M.B., Negrato C.A. (2016). Adherence to insulin therapeutic regimens in patients with type 1 diabetes. A nationwide survey in Brazil. Diabetes Res. Clin. Pract..

[9] Al-Qerem W., Jarab A., Hammad A., Eberhardt J., Alasmari F., Alkaee S.M., Alsabaa Z.H., Al-Ibadah M. (2024). The association between health literacy and quality of life of patients with type 2 diabetes mellitus: A cross-sectional study. PLoS ONE.

[10] Alhaddad J.A., Alshakes N.A., Aljasim M.N. (2023). Quality of Life Among Children with Type 1 Diabetes Mellitus in Alahsa: A Cross-Sectional Study. Cureus.

[11] Pintus D., Ng S.M. (2019). Freestyle libre flash glucose monitoring improves patient quality of life measures in children with Type 1 diabetes mellitus (T1DM) with appropriate provision of education and support by healthcare professionals. Diabetes Metab. Syndr..

[12] Ministy of Health Bahrain Diabetes. https://www.moh.gov.bh/Services/Diabetes?lang=en.

[13] International Diabtes Federation Bahrain Diabetes Report 2000–2045. https://www.diabetesatlas.org/data/en/country/15/bh.html.

[14] Yoldi-Vergara C., Conget-Donlo I., Cardona-Hernandez R., Ramon-Krauel M. (2024). Influence of socioeconomic factors on glycemic control, therapeutic adherence and quality of life in children and adolescents with type 1 diabetes. Endocrinol. Diabetes Nutr. (Engl. Ed.).

[15] Varni J.W., Delamater A.M., Hood K.K., Raymond J.K., Chang N.T., Driscoll K.A., Wong J.C., Yi-Frazier J.P., Grishman E.K., Faith M.A. (2018). PedsQL 3.2 Diabetes Module for Children, Adolescents, and Young Adults: Reliability and Validity in Type 1 Diabetes. Diabetes Care.

[16] Majedah A.-R., AlOtaibi F., AlMahdi M., AlKandari K. (2012). Reliability and validity of the Arabic version of the PedsQLTM 4.0 generic ore scales and PedsQLTM 3.0 diabetes module. J. Diabetes Mellit..

[17] Nakamura N., Sasaki N., Kida K., Matsuura N., Study Group of Health Sciences R. (2010). Health-related and diabetes-related quality of life in Japanese children and adolescents with type 1 and type 2 diabetes. Pediatr. Int..

[18] Lukacs A., Varga B., Kiss-Toth E., Soos A., Barkai L. (2014). Factors influencing the diabetes-specific health-related quality of life in children and adolescents with type 1 diabetes mellitus. J. Child. Health Care.

[19] Lafontaine S., Mok E., Frei J., Henderson M., Rahme E., Dasgupta K., Nakhla M. (2023). Associations of Diabetes-related and Health-related Quality of Life with Glycemic Levels in Adolescents with Type 1 Diabetes Preparing to Transition to Adult Care. Can. J. Diabetes.

[20] Alshammari F., Ansari M., Khan K.U., Neupane D., Hussain A., Anwar S., Alshammari B., Alrasheeday A., Jamshed S., Sapkota B. (2024). Health-related quality of life among people with diabetes: A cross-sectional study in Hail region, Saudi Arabia. PLoS ONE.

[21] Alfaleh A., Alkattan A., Alzaher A., Alhabib D., Alshatri A., Alnamshan A., Almalki O., Almutairi L., Khairat M., Sagor K. (2023). Quality of life among schoolchildren with type 1 diabetes mellitus and the satisfaction of their guardians towards school health care in Saudi Arabia. Diabetes Res. Clin. Pract..

[22] Wilson V. (2008). Barriers to effective communication between patients using insulin pump therapy technology to enable intensive diabetes self-management and the health professionals providing their diabetes care. J. Assist. Technol..

[23] Ghazanfar H., Rizvi S.W., Khurram A., Orooj F., Qaiser I. (2016). Impact of insulin pump on quality of life of diabetic patients. Indian J. Endocrinol. Metab..

[24] Lasaite L., Dobrovolskiene R., Danyte E., Stankute I., Razanskaite-Virbickiene D., Schwitzgebel V., Marciulionyte D., Verkauskiene R. (2016). Diabetes distress in males and females with type 1 diabetes in adolescence and emerging adulthood. J. Diabetes Complicat..

[25] Galler A., Tittel S.R., Baumeister H., Reinauer C., Brosig B., Becker M., Haberland H., Hilgard D., Jivan M., Mirza J. (2021). Worse glycemic control, higher rates of diabetic ketoacidosis, and more hospitalizations in children, adolescents, and young adults with type 1 diabetes and anxiety disorders. Pediatr. Diabetes.

[26] Yilmaz Karaman I.G., Altinoz A.E., Aydin Buyruk B., Yorulmaz G., Kosger F., Kirel B. (2023). Comparison of anxiety, stress, and social support levels of female patients with type 1 diabetes and mothers whose children have type 1 diabetes. J. Diabetes Metab. Disord..

[27] Luo D., Wang Y., Cai X., Li R., Li M., Liu H., Xu J. (2022). Resilience Among Parents of Adolescents With Type 1 Diabetes: Associated With Fewer Parental Depressive Symptoms and Better Pediatric Glycemic Control. Front. Psychiatry.

[28] Samardzic M., Tahirovic H., Popovic N., Popovic-Samardzic M. (2016). Health-related quality of life in children and adolescents with type 1 diabetes mellitus from Montenegro: Relationship to metabolic control. J. Pediatr. Endocrinol. Metab..

[29] Levesque A.R., MacDonald S., Berg S.A., Reka R. (2021). Assessing the Impact of Changes in Household Socioeconomic Status on the Health of Children and Adolescents: A Systematic Review. Adolesc. Res. Rev..

[30] Rohilla L., Gujjar N., Kaur G., Walia P., Dayal D. (2022). Financial burden for families of children with type 1 diabetes: A cross-sectional survey from North India. Diabetol. Int..

[31] Bittner J.M.P., Gilman S.E., Zhang C., Chen Z., Cheon B.K. (2023). Relationships between early-life family poverty and relative socioeconomic status with gestational diabetes, preeclampsia, and hypertensive disorders of pregnancy later in life. Ann. Epidemiol..

[32] Ozguven Oztornaci B., Ardahan Akgul E., Yanar N., Akyol S., Yetim P., Bas G., Yildirim Sari H., Dundar B.N. (2024). The Effect of Parental Collaboration on Diabetes Self-Efficacy, Quality of Life and HbA1c Level in Adolescents Diagnosed with Type 1 Diabetes. J. Clin. Res. Pediatr. Endocrinol..

[33] Alsahli M.A., Alalwan A., Aburisheh K.H., Alarifi F.F., Alshaya H.M., Alkholaif A.F., Shadid A.M., Alsahli S.A., Alsahly A.A., Alkhalifah M.K. (2024). Assessing satisfaction, quality of life, and HbA1c changes in type 1 diabetes patients who are using freestyle libre glucose monitoring. J. Fam. Med. Prim. Care.

[34] Alipour A., Zare H., Poursharifi H., Aerab Sheibani K., Ardekani M.A. (2012). The intermediary role of self-efficacy in relation with stress, glycosylated haemoglobin and health-related quality of life in patients with type2 diabetes. Iran J. Public Health.

[35] Oakley N.J., Kneale D., Mann M., Hilliar M., Dayan C., Gregory J.W., French R. (2020). Type 1 diabetes mellitus and educational attainment in childhood: A systematic review. BMJ Open.

[36] Martens T., Beck R.W., Bailey R., Ruedy K.J., Calhoun P., Peters A.L., Pop-Busui R., Philis-Tsimikas A., Bao S., Umpierrez G. (2021). Effect of Continuous Glucose Monitoring on Glycemic Control in Patients With Type 2 Diabetes Treated With Basal Insulin: A Randomized Clinical Trial. JAMA.

